# CM-YOLO: A Multimodal PCB Defect Detection Method Based on Cross-Modal Feature Fusion

**DOI:** 10.3390/s25134108

**Published:** 2025-06-30

**Authors:** Haowen Lan, Jiaxiang Luo, Hualiang Zhang, Xu Yan

**Affiliations:** 1 School of Automation Science and Engineering, South China University of Technology, Guangzhou 510640, China; 202230464243@mail.scut.edu.cn (H.L.); 202330335051@mail.scut.edu.cn (X.Y.); 2 School of Software Engineering, South China University of Technology, Guangzhou 510640, China; 202330333731@mail.scut.edu.cn

**Keywords:** PCB defect detection, multimodal, feature fusion

## Abstract

By integrating information from RGB images and depth images, the feature perception capability of a defect detection algorithm can be enhanced, making it more robust and reliable in detecting subtle defects on printed circuit boards. On this basis, inspired by the concept of differential amplification, we propose a novel and general weighted feature fusion method within the YOLO11 dual-stream detection network framework, which we name CM-YOLO. Based on the differential amplification approach, we introduce a Differential Amplification Weighted Fusion (DAWF) module, which separates multimodal features into common-mode and differential-mode features to preserve and enhance modality-specific characteristics. Then, the SE-Weighted Fusion module is used to fuse the common-mode and differential-mode features.In addition, we introduce a Cross-Attention Spatial and Channel (CASC) module into the detection network to enhance feature extraction capability. Extensive experiments show that the proposed CM-YOLO method achieves a mean Average Precision (mAP) of 0.969, demonstrating the accuracy and effectiveness of CM-YOLO.

## 1. Introduction

The PCB (Printed Circuit Board) is an essential component in electronic devices, widely used in consumer electronics, computers, automotive electronics, medical devices, and other fields. The quality of the PCB is closely related to the overall product quality. However, with the significant advancements in modern technology, the design and manufacturing processes of PCBs have become increasingly complex, which can lead to various defects. Therefore, a reliable defect detection method can effectively ensure the quality of PCBs [1,2]. Many industrial studies have also focused on PCB defect detection [3,4,5].

In recent years, with the rapid development of deep learning (DL), convolutional neural networks (CNNs) have emerged as a novel and powerful feature extraction method, greatly enhancing object detection capabilities. Current object detection algorithms are mainly divided into single-stage and two-stage approaches [6,7]. For instance, ref. [8] proposed a two-stage detection method based on Faster R-CNN for vehicle detection, where candidate regions are first generated and then classified, dividing the detection process into two stages. Pan [9] and others proposed a defect object detection algorithm based on YOLO, which does not require a separate region proposal step and simultaneously predicts object categories and locations through a single network. At present, many studies on object detection focus on defect detection [10,11,12,13,14,15]. Studies [16,17], based on two-stage methods such as Faster R-CNN, have achieved excellent performance in PCB defect detection. Study [18] employed a single-stage method for detecting defects on PCB boards.

Most traditional surface defect detection methods belong to the category of single-modality 2D image object detection tasks in computer vision. For example, an algorithm for PCB defect detection, named PCB-YOLO, was introduced based on the YOLOv5 framework [19]. Du et al. [20] improved YOLO’s network by adding Mobile Inverted Bottleneck Convolution (MBConv) modules, Convolutional Block Attention Modules (CBAM) [21], Bidirectional Feature Pyramid Networks (BiFPN), and depth convolutions, resulting in YOLO-MBBi for PCB surface defect detection. However, because RGB images can only capture surface color and texture information, they are susceptible to interference when detecting subtle, hidden, or light-affected defects. In practical applications, slight lifting may be difficult to detect due to similar colors or reflection issues, reducing detection accuracy. Additionally, the depth information or material properties of defects cannot be directly obtained from 2D images. In fact, multimodal information processing is more aligned with biological or human cognitive patterns. Multimodal representations of the same target are more comprehensive and complete compared to single-modality representations, and multimodal detection can combine multiple types of information, such as RGB images, depth maps, and infrared images. Li et al. [22] proposed a depth geometric convolution operator (DGConv) to extract geometric information from depth maps for detection. Depth maps can provide three-dimensional structural information of lifted areas, helping to identify corresponding defects, thus improving the accuracy and reliability of defect detection.

The main challenge in PCB defect detection lies in identifying subtle surface defects. The core issue is that single-modal information lacks sufficient descriptiveness, and defects often exist in regions where the feature distributions of normal and abnormal samples overlap, making them difficult to distinguish accurately. Multimodal defect detection methods offer an effective solution to this problem; however, while multimodal features contain critical information, they also introduce significant noise. If we merely adopt simple fusion methods such as addition, multiplication, or concatenation, this increases the difficulty of cross-modal learning in the network, leading to performance degradation. Many studies have explored multimodal approaches [23,24,25,26,27]. In our PCB multimodal detection, 2D features generally represent color, texture, and shape information, whereas 3D features capture surface height variations and structural details. Effectively utilizing both 2D and 3D feature information remains a key focus of current research.

**Figure 1:**
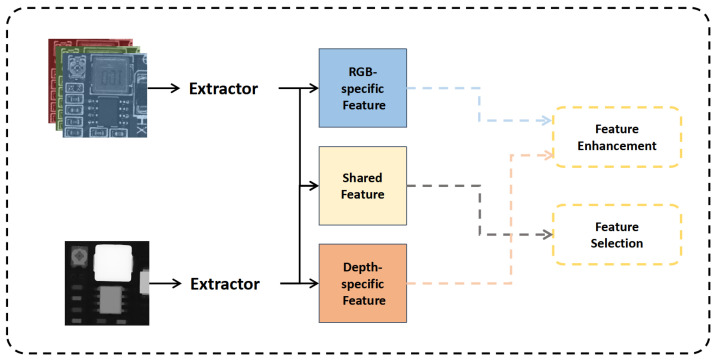
Selecting common-modal and differential-modal features from the multimodal features.

To address these issues, we propose a multimodal detection method based on the YOLO11 (You Only Look Once version 11) framework, incorporating cross-modal spatial and channel attention, as well as a common-mode–differential-mode weighted feature fusion approach inspired by differential amplifier circuits. This method aims to enhance multimodal feature representation, improve detection accuracy, and reduce information redundancy. Our feature fusion strategy, as illustrated in Figure 1, divides the features into common-mode and differential-mode components, which are then adaptively fused.

To summarize, our main contributions are as follows:

(1) We propose a dual-stream feature extraction network for defect detection. Based on the latest detection model, YOLO11, we extend it to a dual-stream fusion network that integrates RGB and depth images, named Cross-Modal YOLO (CM-YOLO).
(2) We designed the Cross-modal Attention for Space and Channel (CASC) module. By incorporating the CASC attention module into our dual-stream feature extraction network, we combine channel attention and spatial attention, thereby enhancing the feature extraction capability.
(3) We have introduced a feature fusion module based on the concept of differential amplification into our dual-stream feature extraction network, which we name the Differential Amplification Weighted Fusion (DAWF) module. It divides feature information into shared and specific components while adaptively adjusting weights based on different defect categories using the SE attention mechanism. Finally, the features are fused, enabling the network to effectively utilize both 2D and 3D features.

The structure of this paper is organized as follows: Section 2 reviews recent research in the field, Section 3 provides a detailed explanation of the method we propose, Section 4 presents the experimental results, and Section 5 concludes the paper.

## 2. Related Work

This section introduces related research on printed circuit board (PCB) image defect detection, including PCB defect detection and multimodal object detection studies.

### 2.1. PCB Defect Detection

Surface defect detection methods based on deep learning mainly focus on obtaining information such as defect type, defect location, and defect specifications, corresponding to visual tasks such as defect classification, localization (detection), and segmentation. Methods [28,29] have applied deep learning to image defect detection, achieving excellent results. Currently, surface defect detection is generally divided into defect detection and anomaly detection tasks. Defect detection is a multi-class problem in pattern recognition, where labeled defect data is used to train a supervised object detection model, enabling defect classification and localization. In contrast, anomaly detection is a binary classification problem that focuses on whether the tested sample is normal. It uses normal sample data for unsupervised training to accurately characterize normal samples and performs anomaly judgment and region segmentation by comparing the differences between normal and defect samples.

To address PCB defect detection, advanced deep networks based on CNNs are typically trained on large-scale, high-quality public natural image datasets. Compared to many other methods, CNN models have demonstrated strong performance [30] and have been applied to PCB defect detection, such as DeepPCB [31]. Due to YOLO’s compact network structure, fast execution speed, and outstanding detection performance, many studies have applied YOLO to PCB defect detection. Ref. [32] proposed a novel improved YOLOv3 method [33], which introduced the CBAM module and GIoU loss function [21,34] to further enhance the network’s performance for detecting PCB soldering defects. Tang et al. [19] proposed a YOLOv5-based defect detection method that integrates transfer learning and attention mechanisms to improve the accuracy of PCB detection. Yuan et al. [35] proposed the YOLO-HMC model, which aggregates semantic information more efficiently and accurately by using a hybrid direction-aware convolutional network (HorNet), multi-convolution block attention module (MCBAM), and content-aware feature reorganization (CARAFE), thereby enhancing the detection capability of PCB micro-defects.

As a single-stage network, the YOLO series features a simple structure, making it easier to modify. With the continuous iteration of the YOLO series, this paper adopts the latest version, YOLO11, as the basic framework for multimodal detection research. YOLO11 has been improved into a dual-stream detection network that extracts both 2D and 3D features and fuses the multimodal features for defect detection in PCBs.

### 2.2. Multimodal Object Detection

**Figure 2:**
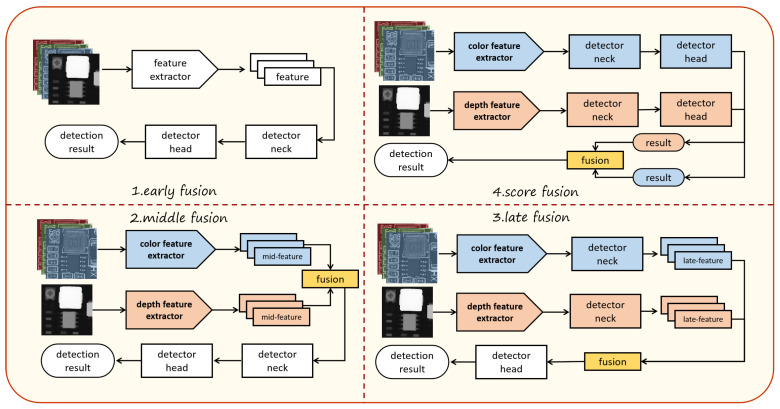
The four common fusion methods in current multimodal fusion.

The essential aspect of multimodal object detection is the method of fusion. Based on the fusion stages outlined in [24], existing feature fusion techniques can be categorized into four types: early fusion, middle fusion, late fusion, and score fusion, as illustrated in Figure 2. Work in [24] indicates that intermediate fusion performs the best. In GFD-SSD [36], two novel GFUs are proposed to facilitate the fusion of intermediate layer feature maps from two SSDs. CIAN [37] adopts a cross-modal attention method to unify the convergence of feature maps during intermediate fusion, which is further enhanced by Context Enhancement Blocks (CEBs). Therefore, this paper adopts an intermediate fusion strategy to fuse RGB and depth image features.

In multimodal object detection tasks, the specific design and functionality of the fusion module are critical for capturing complementary information between RGB and depth images. To capture the discriminative object features in multispectral images, MSDS-RCNN [38] is the first work to use semantic segmentation to guide multispectral object detection via multi-task learning. The design of the fusion module should adopt a reasonable approach to efficiently fuse features from 2D and 3D images while retaining and enhancing effective features and suppressing redundant features, thereby improving detection efficiency.

Currently, the mainstream multimodal image fusion methods can be divided into three categories:

(1) **CNN-based Fusion Methods**: These methods mainly utilize the powerful feature extraction capabilities of Convolutional Neural Networks (CNNs) to fuse information from different modal images, generating more comprehensive and richer feature representations. For example, in [39], Zhang proposed the IFCNN method, which adopts a CNN structure aimed at providing a general framework for various image fusion tasks. The CNN structure is typically composed of multiple convolutional layers, enabling IFCNN to handle multi-scale information in images, thereby helping to preserve the details and overall structure of the image.
(2) **Encoder-Decoder-based Multimodal Image Fusion Methods**: This approach uses a deep learning network (encoder) to extract and fuse features from different modal images and then reconstructs a high-quality fused image through a decoder. In [40], Li et al. proposed the DenseFuse method, which introduces the idea of dense connections, enabling the network to better capture the relationships between features when handling fusion tasks of infrared and visible light images.
(3) **Generative Adversarial Network (GAN)-Based Fusion Methods**: These methods treat the fused image as a generator and design a discriminator to evaluate it, thereby improving the quality of the fused image. A pioneering work applying this technology in multimodal detection is [41], where the cm-SSFT algorithm was proposed. It combines shared modal information with modality-specific features and uses a Shared-Specific Transmission Network (SSTN) for information propagation and complementary learning, thus effectively enhancing the discriminative power and complementarity of the features, overcoming the limitations of traditional methods that only focus on shared features.

In the original YOLO11 model, the fusion methods used, such as concatenation, fail to effectively utilize the complementary semantic information between RGB and depth images. In this paper, we designed a cross-modal fusion method based on the differential amplification approach. This method performs the weighted fusion of extracted common-mode and differential-mode features, achieving feature differentiation across modalities and fusing multi-semantic information. Moreover, it integrates the SE attention mechanism and BiFPN to form our fully adaptive weighted fusion method.

## 3. Methodology

### 3.1. CM-YOLO

Compared to two-stage detection methods, the YOLO series algorithms offer faster end-to-end inference speed, making them suitable for efficient real-time PCB defect detection. Additionally, YOLO is more adaptable to small-scale datasets and lightweight models. Its simple architecture is easy to modify and optimize, and it improves the detection of small targets. YOLO has low computational overhead on embedded or edge devices, enabling efficient deployment while maintaining a balance between detection accuracy and inference speed, making it an ideal choice for industrial scenarios.

**Figure 3:**
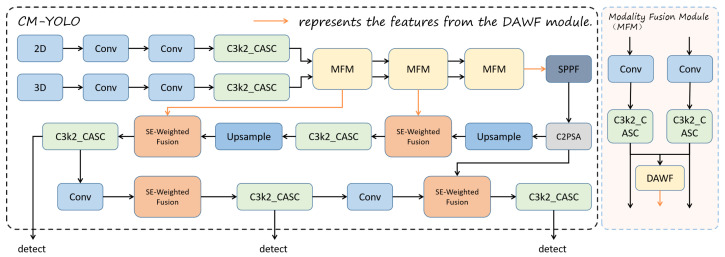
Our Cross-Modal YOLO.

As shown in Figure 3, based on the concept of cross-modality, we modify the structure of YOLO11 into a dual-stream feature extraction network by replacing the original YOLO backbone with two feature extraction branches, which extract features from RGB images and depth images. We introduce the CASC module and the DAWF module to effectively extract and fuse features. This forms our proposed CM-YOLO.

### 3.2. Cross Attention of Space and Channel Module

The C3K2 module (C3 with Kernel-2) is an important improvement in YOLO11’s backbone and neck structure. Compared to the CF2 (Cross-Stage Fusion) module in YOLOv8, the C3K2 module adopts a new convolutional combination strategy and introduces the Kernel-2 (K2) structure. This enhances hierarchical feature extraction, optimizes cross-channel information interaction, and improves feature extraction capability and information flow.

**Figure 4:**
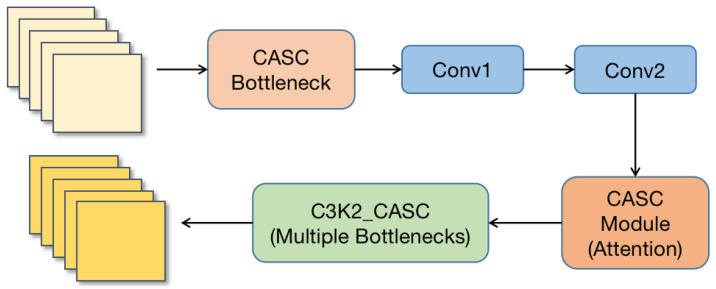
The C3K2 feature extraction module based on CASC modification.

We propose a Cross-Modal Spatial and Channel Attention module, which effectively enhances the correlation between features, thereby improving the accuracy of defect detection. To integrate CASC into the dual-stream feature extraction network, we combine the CASC module with the C3K2 module, replacing the original bottleneck structure to strengthen the model’s feature extraction capability. This forms the C3K2_CASC module, as shown in Figure 4.

The CASC module combines local and global convolution operations to compute spatial and channel attention, leveraging the attention mechanism to better capture important features while suppressing irrelevant information. With this integration, the C3K2 module not only retains its original computational efficiency but also improves its ability to model image details, enabling the model to perform more effectively and accurately in complex tasks.

**Figure 5:**
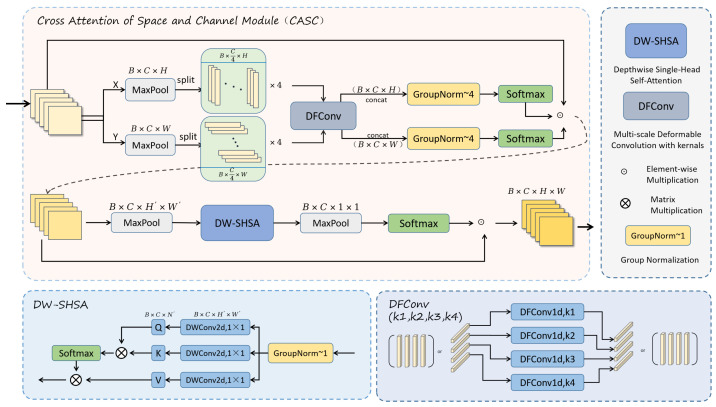
Our Cross-modal Attention for Space and Channel module.

Shown in Figure 5, our CASC module is mainly divided into a spatial attention module and a channel attention module. In our work, we let the input features be $X\in R^{B\times C\times H\times W}$. The input feature tensor has a shape of $X\in R^{B\times C\times W\times H}$, where *B*, *C*, *W*, and *H* denote the batch size, number of channels, width, and height. We divide the feature into two subfeatures along the height $X\in R^{B\times C\times W}$ and width $X\in R^{B\times C\times H}$. We then divide these two sets of features into four separate sub-features:

(1) $$X_{i}=X_{A}\left[:,\left(\frac{C}{4}\right)\times \left(i-1\right):\left(i\times \frac{C}{4}\right),:,:\right],\;X_{A}\in \left(X_{H},X_{W}\right)$$

For the four groups of sub-features $X_{i}$, we design a DFConv module, which includes four adaptive convolution kernels for performing one-dimensional depthwise convolutions. Each kernel processes one of the four groups of sub-features, and the convolution kernels can adaptively adjust their parameters according to the characteristics of the input data. They are capable of automatically selecting the most appropriate kernel size at different locations, thereby extracting fine-grained details more accurately. After passing through the DFConv layer, the convolved features are denoted as ${\hat{X}}_{i}$. Subsequently, the four groups of sub-features are concatenated along the height and width dimensions, which correspond to the spatial dimensions. After group normalization and softmax activation, the resulting feature is denoted as F. Finally, feature F is multiplied with the original feature X to obtain the final feature $X_{F}$.

The formula is shown below:

(2) $${\hat{X}}_{i}=\mathrm{Conv}1d\left(X_{i}\right)$$

(3) $$F=\mathrm{softmax}\left(\mathrm{GN}\left(\mathrm{Concat}\left({\hat{X}}_{i}\right)\right)\right),\;X_{F}=X\times F$$

In the channel attention features, we first use max pooling to preprocess the feature $X_{F}$, making it more sensitive to fine details while reducing noise in the image, resulting in the feature $X_{C}$. To better interact with the sub-channels of spatial attention, we adopt a simpler and more efficient single-head self-attention mechanism, which we name the DW-SHSA module. In the DW-SHSA module, the feature $X_{C}$ is first group-normalized, followed by the computation of the three attention vectors Q, K, and V. Then, the attention mechanism is performed based on Q, K, and V to obtain the feature $X_{A}$. The attention feature $X_{A}$ is subsequently processed using max pooling, and attention weights are obtained through softmax. Finally, the result is multiplied with the original feature $X_{C}$ to generate the final output $X_{\mathrm{out}}$.

Our self-attention mechanism is computed along the channel dimension, meaning that

$$Q,K,V\in R^{B\times C\times N},\;\mathrm{where}\;N=H\times W.$$

This mechanism dynamically adjusts the weight of each channel, enhancing the features of important channels while suppressing irrelevant ones. After global pooling and softmax activation, the final output features can more accurately capture key information in the image. This design not only improves the inter-channel relationships and enhances the accuracy of feature selection but also ensures computational efficiency, increasing the model’s adaptability and robustness to different inputs.

The mathematical expression is given as follows:

(4) $$X_{C}=\mathrm{MaxPool}\left(X_{F}\right)$$

(5) $$F={\mathrm{DWConv}2d}_{\left(1,1\right)}^{C\to C}$$

(6) $$Q=F^{Q}\left(X_{C}\right),\;K=F^{K}\left(X_{C}\right),\;V=F^{V}\left(X_{C}\right)$$

(7) $$X_{A}=\mathrm{Atten}\left(Q,K,V\right)$$

(8) $$X_{\mathrm{out}}=X_{C}\times \mathrm{softmax}\left(\mathrm{MaxPool}\left(X_{A}\right)\right)$$

### 3.3. Differential Amplification Weighted Fusion (DAWF)

In analog electronics, differential amplification circuits are widely used. They can effectively suppress common-mode signals (such as noise) and amplify only the differential signals (the difference between two inputs). Applying the method of differential amplification in our feature fusion allows for the effective extraction and distinction of common-mode and differential-mode features in 2D and 3D data. Through the addition operation, common-mode features between the two modalities can be retained. These features usually contain cross-modal shared information, representing the consistency between different modalities. On the other hand, the subtraction operation helps to extract differential-mode features, which represent unique information between each modality and aid in capturing modality-specific differences. This enables the model to better learn the shared features between the two modalities. The extraction of differential-mode features helps the model to focus on the unique characteristics of each modality, avoiding over-reliance on a single modality, ultimately improving the accuracy and performance of the task. This approach has significant advantages, especially in small object detection, complex scene analysis, and multimodal data fusion tasks.

**Figure 6:**
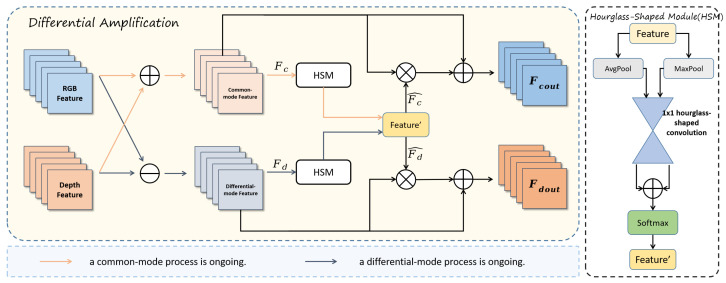
The cross-modal feature selection module based on the differential amplification concept.

Our DAWF module is mainly divided into two parts: cross-modal feature extraction and SE-weighted fusion, shown in Figure 6.

In the cross-modal feature extraction part, we simultaneously extract both common-mode and differential-mode features.We perform operations on the features by adding the two modality features $F_{R}$ and $F_{D}$ to obtain the common-mode feature $F_{c}$, which retains the shared information of both modalities. Subtracting the two features yields the differential-mode feature $F_{d}$. Then, our Hourglass-Shaped Module (HSM) processes these two sets of features. Global average pooling and global max pooling are applied to the common-mode feature $F_{c}$ and differential-mode feature $F_{d}$, resulting in features $F_{1}$ and $F_{2}$. Specifically, $F_{1}$ contains the max-pooled common and differential features ($F_{1c}$, $F_{1d}$), while $F_{2}$ contains the average-pooled common and differential features ($F_{2c}$, $F_{2d}$). The two sub-features of each modality are further processed through a shared convolution layer, which consists of a 1 × 1 hourglass-shaped convolutional network. The common modality sub-features $F_{1c}$ and $F_{2c}$ are passed through this convolution to obtain the feature $F_{3c}$, and, similarly, the differential features are processed to otain $F_{3d}$. These are then activated using the softmax function to obtain the attention weights $\overset{\wedge }{F_{c}}$ and $\overset{\wedge }{F_{d}}$, which are then multiplied by the original common-mode and differential-mode features, respectively. The processed features are concatenated with the original common-mode and differential-mode features to form two cross-modal features $F_{cout}$ and $F_{dout}$. These two features are then fused using SE-Weighted Fusion to obtain the final fused feature.

The mathematical formulation is given as follows:

(9) $$F_{c}=F_{R}+F_{D}$$

(10) $$F_{d}=F_{R}-F_{D}$$

(11) $$\left(F_{1}c,F_{1}d\right)=Avgpool\left(F_{c},F_{d}\right)$$

(12) $$\left(F_{2}c,F_{2}d\right)=Maxpool\left(F_{c},F_{d}\right)$$

(13) $$\left(F_{3}c,F_{3}d\right)=Shconv\left(F_{1}c,F_{1}d\right)+Shconv\left(F_{2}c,F_{2}d\right)$$

(14) $$\left(\overset{\wedge }{F_{c}},\overset{\wedge }{F_{d}}\right)=Soft\max\left(F_{3}c,F_{3}d\right)$$

(15) $$F_{cout}=F_{c}\times \overset{\wedge }{F_{c}}+F_{c},F_{dout}=F_{d}\times \overset{\wedge }{F_{d}}+F_{d}$$

By using the cross-modal feature selection module based on the differential amplification concept, we can separate the features into shared and specific features, thereby enhancing the model’s ability to learn cross-modal features. This enables the model to better perceive small objects from both 2D and 3D dimensions, ultimately improving the accuracy of PCB defect detection.

**Figure 7:**
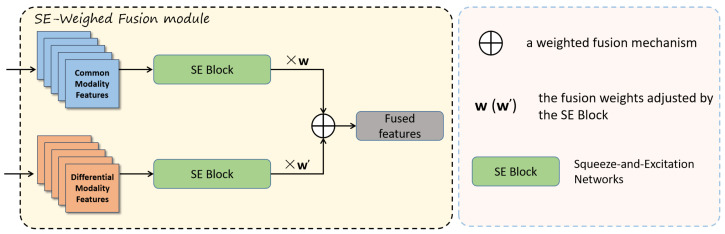
SE-Weighted Fusion module.

The second part of the DAWF module is the SE-Weighed Fusion module, shown in Figure 7. Inspired by the BiFPN weighted fusion technique, it integrates the SE (Squeeze-and-Excitation) attention mechanism to dynamically adjust the importance of feature channels. This approach effectively combines feature maps from different modalities or scales, thereby improving the overall performance of the model. By leveraging the channel-wise attention mechanism of the SE module and adaptively weighting different input features, this method enhances the model’s ability to capture key information while ensuring efficient cross-scale feature aggregation.

In the SE-Weighted Fusion module, a weighted fusion mechanism is employed to combine the two inputs, where the weights are controlled by the learned parameter *w*. This mechanism ensures that the contribution of each input is adaptively adjusted. The weighted fusion is computed using the following formula:

(16) $$F_{\mathrm{fused}}=\frac{w_{1}\cdot F_{cout}+w_{2}\cdot F_{dout}}{w_{1}+w_{2}+\epsilon }$$

The term ϵ is a small constant added to the denominator to prevent division by zero and ensure numerical stability.

On the weighted feature maps, SE Block is applied separately to each of the two inputs to perform channel-level attention adjustment. Each SE Block generates appropriate channel weights based on the characteristics of the input channels, selectively enhancing important channels while suppressing irrelevant ones. After weighting and attention adjustment, the two processed feature maps undergo a BiFPN-inspired fusion strategy, where cross-scale feature information is adaptively aggregated. Finally, the fused feature maps are concatenated to form the final output, effectively enhancing the representation of multi-scale and cross-modal information.

### 3.4. Training Strategy and Loss Function

During the training process, we train our designed modules on the YOLO architecture. Instead of evaluating the effectiveness of each module in isolation, we assess the overall model performance by evaluating the quality of the end-to-end outputs. Consequently, we adjust the parameters through the three loss functions (box loss, cls loss, dfl loss) of YOLO11.

#### 3.4.1. The Box Loss

The goal of this loss function is to make the predicted bounding boxes as close as possible to the true bounding boxes. By minimizing this loss function, the YOLO algorithm learns how to accurately predict the position and size of objects. Boxloss is essential in the training process as it serves multiple critical purposes. It enables precise localization by minimizing the loss associated with the center point coordinates and the dimensions of the bounding box, thereby allowing the model to accurately predict the position and size of objects. Additionally, by applying the square root to the width–height loss, it effectively balances the optimization for targets of varying sizes, ensuring that smaller objects are not overlooked. Furthermore, the well-designed boxloss contributes to stable training, preventing issues like gradient explosion or vanishing gradients that could otherwise disrupt the learning process. The loss function is expressed as

$$\mathrm{Box}\;\mathrm{Loss}=\lambda_{\mathrm{coord}}\sum_{i=0}^{S^{2}}\sum_{j=0}^{B}1_{ij}^{\mathrm{obj}}\left[{\left(x_{i}-{\hat{x}}_{i}\right)}^{2}+{\left(y_{i}-{\hat{y}}_{i}\right)}^{2}\right]$$

(17) $$+\lambda_{\mathrm{coord}}\sum_{i=0}^{S^{2}}\sum_{j=0}^{B}1_{ij}^{\mathrm{obj}}\left[{\left(\sqrt{w_{i}}-\sqrt{{\hat{w}}_{i}}\right)}^{2}+{\left(\sqrt{h_{i}}-\sqrt{{\hat{h}}_{i}}\right)}^{2}\right]$$

where $\lambda_{\mathrm{coord}}$ is used to adjust the proportion of coordinate loss in the total loss. YOLO divides the image into an S×S grid. *B* defines the number of bounding boxes predicted per grid cell. Additionally, $1_{ij}^{\mathrm{obj}}$ is an indicator function that is 1 if the *j*-th bounding box in the *i*-th grid cell is responsible for predicting the object and is 0 otherwise.The parameters associated with the predicted bounding boxes $\left(x_{i},y_{i},w_{i},h_{i}\right)$ represent the center coordinates and dimensions (width and height) of the predicted box. Correspondingly, $\left({\hat{x}}_{i},{\hat{y}}_{i},{\hat{w}}_{i},{\hat{h}}_{i}\right)$ represent the ground-truth box center coordinates and dimensions.

#### 3.4.2. The Classification Loss

This classification loss function is used to calculate the discrepancy between the model’s predicted probability distribution and the true labels. Specifically, it iterates over all grid cells and computes the sum of the squared differences between the predicted probabilities and the true labels for those cells that contain an object. This ensures that the classification loss is only considered when a grid cell actually contains an object.

(18) $$\mathrm{Classification}\;\mathrm{Loss}=\sum_{i=0}^{S^{2}}1_{i}^{\mathrm{obj}}\sum_{c\in \mathrm{classes}}{\left(p_{i}\left(c\right)-{\hat{p}}_{i}\left(c\right)\right)}^{2}$$

where *S* has the same variable explanation as in the boxloss function and $1_{i}^{\mathrm{obj}}$ indicates whether the *i*-th grid cell contains an object (1 if it does, 0 otherwise). $p_{i}\left(c\right)$ is the probability predicted by the model that the object in the *i*-th grid cell belongs to class *c*. ${\hat{p}}_{i}\left(c\right)$ is the ground-truth label, indicating whether the object in the *i*-th grid cell belongs to class *c*.

#### 3.4.3. The DFL Loss

In object detection tasks, class imbalance is a common issue. The number of samples in certain classes may be much higher than in others, which can lead to the model performing well on common classes during training and poorly on rare classes. Additionally, the detection of small targets and difficult samples is also a challenge, as these targets often have less feature information and can be easily overlooked or misclassified.

To address these issues, YOLO proposes the DFL Loss, which introduces a distribution focal loss to enhance the model’s attention to difficult samples and improve the class imbalance problem.

The DFL Loss is defined as

(19) $$DFL=-\sum_{i=1}^{N}\sum_{c=1}^{C}\;y_{ic}(\alpha (1-p_{ic}{)}^{\gamma })\log(p_{ic})+(1-\alpha )(p_{ic}{)}^{\gamma }\log(1-p_{ic})$$

where *N* denotes the total number of samples and *C* represents the count of distinct categories. $y_{ic}$ represents the actual label for the *i*-th sample, formatted in one-hot encoding, where a single entry is set to 1 and all other entries are 0. $p_{ic}$ denotes the forecasted probability that the *i*-th sample falls into category *c*. *α* serves as the balance coefficient, which is utilized to modulate the weights between positive and negative instances. *γ* is the focus parameter, designed to regulate the level of emphasis given to samples that are challenging to classify.

## 4. Experiments

In this section, we conduct experiments on the defects in the multimodal PCB dataset consisting of RGB and depth images to evaluate the performance of the proposed method.

### 4.1. Experimental Setup

To conduct experiments on our model, we used a depth camera to capture RGB and depth images and constructed a dataset named the Multimodal PCB Dataset. As mentioned in [3], common defect types in industrial applications include short circuits, solder defects, physical damage, insufficient insulation, and component misplacement. To make our dataset as close to real-world industrial scenarios as possible, we included five types of defects: component missing, solder bridging, lifted pin, component shift, and dirt. We conducted experiments on all defect types within this dataset.

Our depth information is obtained using a high-precision 3D camera that integrates structured light with a 25 MP CMOS sensor (Gpixel Changchun Microelectronics Inc., Changchun, China) to deliver a depth resolution of 1280 × 720, a Z-axis repeatability of 165 nm, and a measurement field of 54 mm × 46 mm at less than 6 FPS. This system ensures the stable acquisition of multimodal data, capturing texture, contour, and surface geometry. Specifically, depth data are first extracted in TIFF point cloud files (Tagged Image File Format) and then converted into depth maps. The original RGB images have a resolution of 5312 × 4608 pixels. To standardize the dataset, we perform 3× downsampling and crop the defect regions, resulting in 512 × 512 pixel samples that retain local details and preserve multimodal alignment.

**Figure 8:**
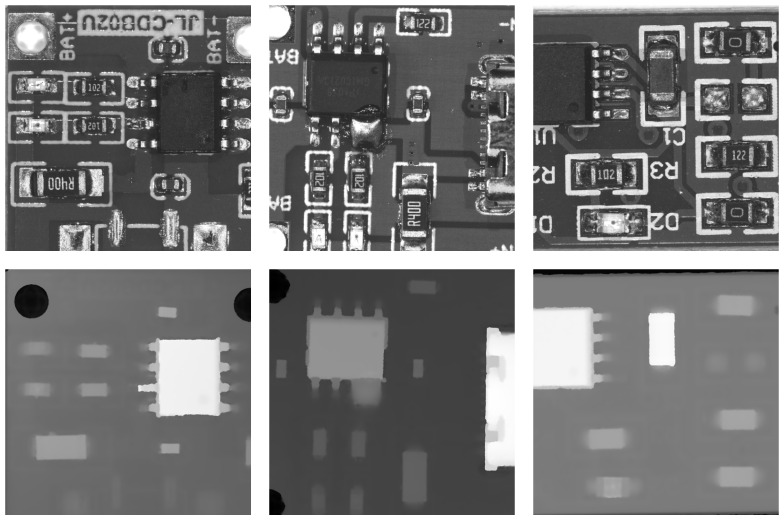
In this set of images, we present our multimodal PCB dataset. The first row shows the images representing the RGB modality, while the second row displays the images representing the depth modality.

The amount of data is shown in Table 1, and sample images from the dataset are illustrated in Figure 8.

Our CM-YOLO detector uses YOLO11-small as the baseline, and all models are trained from scratch. The advantage of YOLO11s lies in its lightweight design and low computational resource requirements, making it highly suitable for low-latency and high-speed PCB defect detection applications. The input image size is 640 × 640, with a batch size of four images. In the experiment, we selected an initial learning rate of $1\times 10^{-2}$ and an SGD optimizer with a momentum of 0.937. The total number of epochs is set to 400. The experimental platform is an Ubuntu 22.04 LTS 64-bit operating system, with a server CPU model of 3rd generation Intel Xeon Platinum 8352 V, and dual GeForce RTX 3080 GPUs. The deep learning framework used is PyTorch 2.4.0.

### 4.2. Experimental Metrics

To evaluate the performance of our CM-YOLO module and compare it with other methods, this paper adopts the following metrics [16]: precision, recall, AP (Average Precision), and mAP@0.5 (mean Average Precision at IoU threshold of 0.5).

(20) $$\mathrm{Precision}=\frac{TP}{TP+FP}$$

(21) $$\mathrm{Recall}=\frac{TP}{TP+FN}$$

(22) $$\mathrm{AP}=\int_{0}^{1}\mathrm{Precision}\left(r\right)\;dr$$

(23) $$\mathrm{mAP}\text{@}0.5=\frac{1}{N}\sum_{i=1}^{N}{\mathrm{AP}}_{i}$$

TP occurs when the model correctly detects a target, FP happens when the model detects a target that does not exist, and FN happens when the model fails to detect an actual target.

### 4.3. Ablation Study

Our ablation experiments were conducted on our PCB dataset. The baseline is the mono-modality detectors with RGB-only and depth-only, which are initialized from YOLO11-small. To verify the performance improvements of structures such as the Two-Stream Network, CASC, and DAWF on the model, ablation experiments were conducted on CM-YOLO. First, we transformed YOLO11s into a two-stream detection network, forming our YOLO11-Multi model. Please note that here we only use a simple concatenation method to fuse the features from the two modalities. Then, we successively added the C3K2 module based on CASC transformation and the DAWF module to verify their contributions to the detection results. Finally, we incorporated both modules into the network and checked for potential mutual interference issues. The experimental results are shown in Table 2.

The experimental results in Table 2 indicate th at, compared to the single-modal image detection of YOLO11, our proposed dual-stream YOLO11 model, by means of cross-modal feature fusion, effectively combines RGB and depth information, achieving an mAP of 0.946, with an approximately 3.9% increase in mAP, which is significantly superior to the single-modal model. As shown in Table 3, the incorporation of 3D information for detection has led to a substantial improvement in the precision of identifying objects with lifted pin, reaching 0.810, while also maintaining a high standard for the detection rates of other categories. These results demonstrate the necessity of dual-stream detection; the fusion of multimodal information enables the model to capture a more comprehensive set of defect features, thereby enhancing the accuracy and robustness of detection.

From Table 2, it can be observed that, compared to the multimodal YOLO11 model without the CASC module, after incorporating the CASC module, the YOLO11-CASC model achieves a precision of 0.861 and a recall of 0.981, while the mAP is further improved to 0.956, which is a 4.9% increase versus the baseline. Simultaneously, as shown in Table 3, while maintaining good detection accuracy for component missing, solder bridging, component shift, etc., the detection precision for lifted pin is further enhanced to 0.867. The results indicate that the CASC module, by integrating spatial and channel attention information, can more accurately locate key regions and features, thereby improving the model’s capability for defect detection.

To demonstrate the importance of the Differential Amplification-based Weighted Feature Fusion module (DAWF), the experimental results presented in Table 2 highlight the performance of the YOLO11-DAWF model. After incorporating the DAWF module, the model shows an improvement in precision to 0.855, a substantial increase in recall to 0.984, and an mAP of 0.958, reflecting a 5.1% improvement compared to the baseline. These findings suggest that the DAWF module, by effectively combining the 2D information from RGB images and the 3D information from depth images using weighted fusion of both common-mode and differential-mode features, enhances the model’s feature representation and detection accuracy. The DAWF module notably boosts the model’s capacity to detect potential defect areas, improving its ability to interpret complex scenarios.

Ultimately, we integrated the CASC module and the DAWF module into the YOLO11 dual-stream detection network, forming our CM-YOLO. The synergy of these two modules enabled the network to achieve optimal detection performance, with a mAP of 0.969, and precision and recall rates of 0.912 and 0.984, respectively. In terms of detection accuracy for each category (see Table 3), the detection accuracy for component shift reached 0.995, lifted pin was 0.913, solder bridging was 0.993, component missing was 0.974, and dirt was 0.968, all demonstrating high detection precision.

### 4.4. Comparison with Other Methods

Table 3 presents the detection results of existing classical methods and our proposed CM-YOLO on the PCB dataset, including the Average Precision (AP) for each category and the overall mean Average Precision (mAP). In terms of overall mAP (0.969) and detection precision across different categories, our model outperforms all classical methods. Compared to YOLOv5, which achieves the best performance among single-modal methods, our CM-YOLO achieves a 3% improvement in mAP. Furthermore, it outperforms our intermediate fusion multimodal approach by 2.3% in mAP, with detection rates for all categories exceeding 90%, achieving the best detection performance.

**Figure 9:**
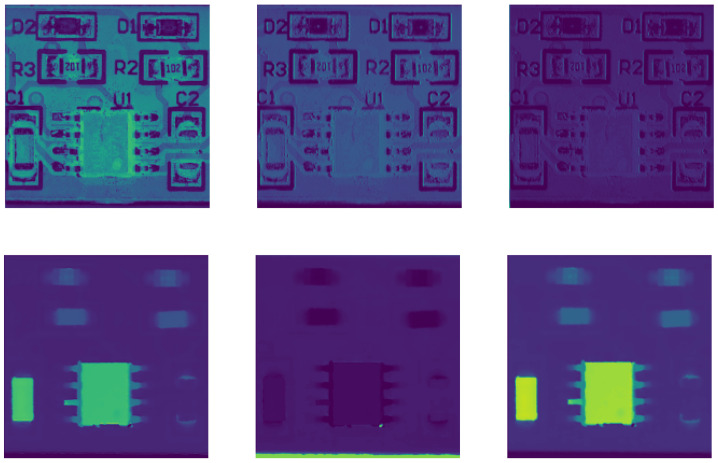
The figure shows the visualization of our feature maps. The first column displays the RGB feature map visualization, and the second column shows the depth map feature map visualization. It can be clearly observed that the RGB images focus more on global two-dimensional features, while the depth images are highly sensitive to three-dimensional information.

Notably, the detection rate for lifted pins reached 91.3%, whereas, in 2D-based methods, the detection performance for lifted pins is significantly lower. This is because 2D PCB defect detection lacks 3D information. As shown in Figure 9, normal pins and lifted pins appear almost identical in 2D images, while they show clear differences in 3D depth maps. This often leads 2D models to mistakenly classify normal pins as lifted pins. This also demonstrates the necessity and effectiveness of our cross-modal detection approach.

To further demonstrate the effectiveness of our method in industrial defect detection, we applied the latest state-of-the-art (SOTA) method, D-Fine [46], to our dataset for comparison. D-Fine significantly improves localization accuracy in object detection by introducing Fine-grained Distribution Refinement (FDR) and Globally Optimal Localization Self-Denosing (GO-LSD). As shown in Table 3, we compare our method with D-Fine. From the overall mean Average Precision (mAP), CM-YOLO achieves 0.969, surpassing D-Fine with RGB input (0.961) and with depth input (0.944), demonstrating the best comprehensive detection performance. In terms of specific categories, CM-YOLO performs better on component missing, solder bridging, and particularly on lifted pin, where it achieves the highest detection accuracy of 91.3%, outperforming D-Fine’s 89.1% (Depth) and 88.2% (RGB). This validates the effectiveness of our multimodal detection approach.

**Figure 10:**
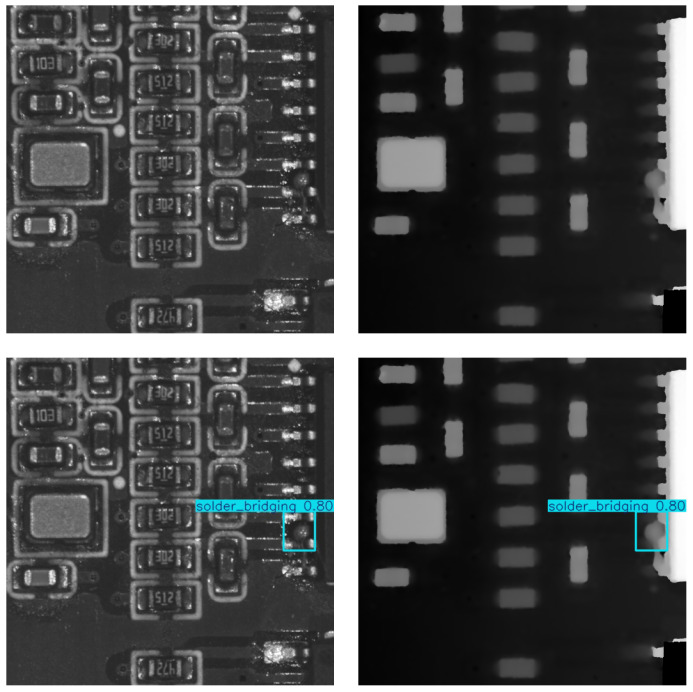
The figure shows a comparison of our detection results. In our baseline method, the detection result for the solder bridging defect is “no detection,” while, in our CM-YOLO method, it is successfully detected. The reason is that the defect’s features are relatively weak, which effectively demonstrates that our CM-YOLO method enhances the model’s ability to extract features.

**Figure 11:**
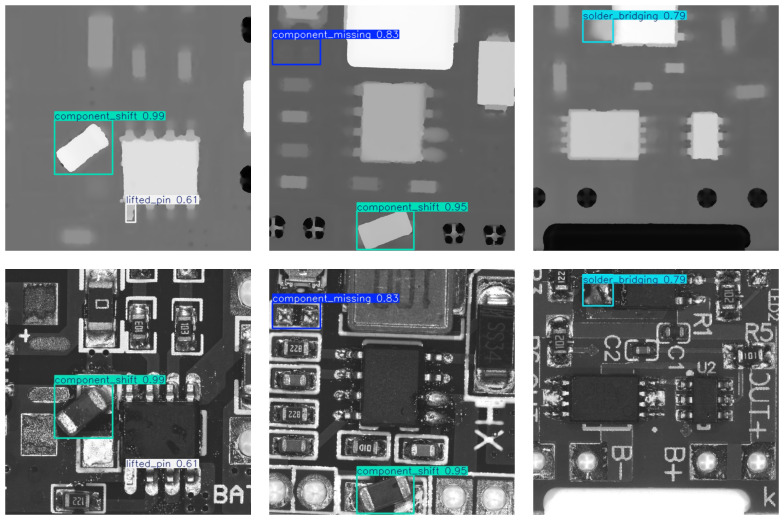
The figure shows the visualization of our detection results.

Figure 10 shows a comparison of detection results between our CM-YOLO and the baseline, while Figure 11 presents example detection results of our CM-YOLO.

We also conducted a comprehensive comparison of several mainstream models in terms of key aspects such as inference speed, model size, and parameter count. The evaluation metrics included total inference time, average inference time per image, frames per second (FPS), model size, and the number of parameters. Specifically, we selected Faster R-CNN as a representative two-stage detection method, YOLOv8 as a representative single-stage detection method, YOLO11 as our baseline model, and D-Fine as the current state-of-the-art (SOTA) method. These four models were compared against our proposed CM-YOLO. The results are summarized in Table 4. It is worth noting that the number of images processed by multimodal models is twice that of unimodal models.

Compared with traditional two-stage detection methods (e.g., Faster R-CNN), our proposed CM-YOLO demonstrates a significant advantage in inference speed, requiring only 22.02 milliseconds per image on average and achieving 45.44 FPS. While maintaining high detection accuracy, CM-YOLO also delivers excellent computational efficiency, with a model size of just 5.20 MB and approximately 2.51 million parameters—considerably smaller than other mainstream models.

Compared to single-stage, single-modal YOLO series models, CM-YOLO maintains a similar level in terms of model size and parameter count. Although it is slightly slower in inference speed, it achieves higher detection accuracy, making it a more balanced choice overall.

Furthermore, compared with the latest SOTA method D-Fine, CM-YOLO shows a significant advantage in both inference time and model size, demonstrating its high practicality and deployment value for real-time industrial defect detection tasks.

To further improve and strengthen the reliability of our experimental results, we conducted five runs of the CM-YOLO method with different random seeds, calculating the precision, recall, and mAP metrics separately. The results are shown in Table 5. The mean precision is 0.9368, with a standard deviation of 0.0114 and a 95% confidence interval of [0.9219, 0.9517]. The mean recall is 0.9685, with a standard deviation of 0.0034 and a 95% confidence interval of [0.9649, 0.9722]. The mean mAP is 0.9708, with a standard deviation of 0.0019 and a 95% confidence interval of [0.9689, 0.9728].

These statistical indicators demonstrate that CM-YOLO achieves high and stable performance under different random initializations. The small standard deviations and narrow confidence intervals indicate low variability in the results, further validating the reliability and practicality of our method, and providing solid experimental support for its real-world application.

**Figure 12:**
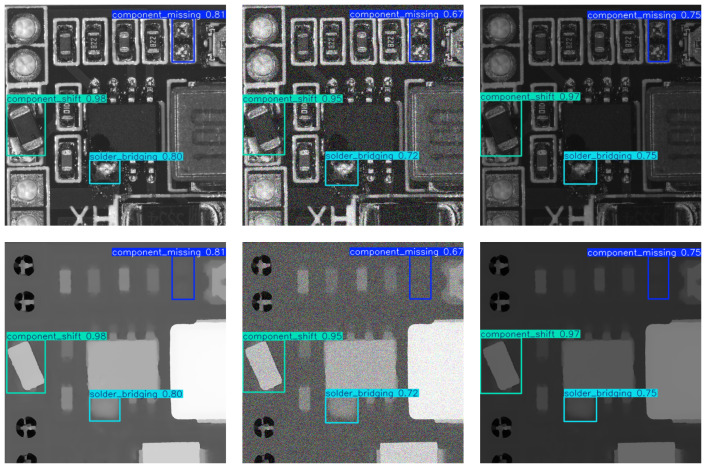
In the figure, the leftmost column shows our normal detection results, the middle column displays the detection results on the dataset with added noise, and the rightmost column presents the detection results on the dataset with reduced brightness. It can be seen that our method still maintains strong detection performance when simulating real industrial inspection scenarios.

To validate the robustness and effectiveness of our proposed method in real-world industrial environments, we conducted experiments by simulating practical conditions. Specifically, we processed the dataset in two ways: (1) reducing the brightness of the images to simulate poor lighting, and (2) adding Gaussian noise to simulate sensor or transmission interference. As shown in Table 6, our CM-YOLO model maintained strong performance under these challenging conditions. On the darkened dataset, the model achieved a precision of 0.933, recall of 0.952, and mAP of 0.959. On the dataset with added noise, it still achieved a precision of 0.880, recall of 0.934, and mAP of 0.946. These results demonstrate that our method remains accurate and reliable even in adverse industrial scenarios, highlighting its practical applicability. The detection results of experiments simulating industrial scenarios are shown in Figure 12. In the future, we will further explore the extension of this method to a wider range of industrial tasks, aiming to provide more reliable detection solutions for real-world production environments.

### 4.5. Comparison on FLIR Dataset

**Figure 13:**
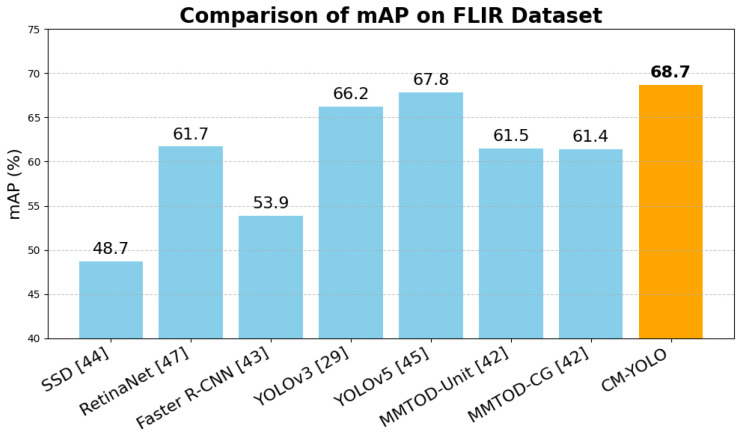
Comparison on FLIR dataset.

Due to the lack of a publicly available multimodal PCB dataset, we further validated the effectiveness of our model by testing it on the FLIR infrared multispectral dataset. This dataset contains a total of 9622 images, which were split into training and test sets in an 80:20 ratio. The dataset includes three categories: person, car, and bicycle. Our experimental results are shown in Figure 13. We primarily compared our model with several classical unimodal models and the multimodal model MMTOD [47]. The experimental results demonstrate that, whether compared with unimodal models or multimodal models, our CM-YOLO model still shows the best performance. This further proves that our method is suitable for most multimodal object detection tasks.

## 5. Conclusions

This paper proposes a CM-YOLO detection method for PCB defect detection based on an improved YOLO11. A Cross-modal Attention for Space and Channel (CASC) mechanism is introduced to modify the C3K2 feature extraction module, enhancing the capability of extracting features from small-sized defects in complex backgrounds. Additionally, we propose a Differential Amplification Weighted Fusion (DAWF) module that fully leverages the complementary features between different modalities, selecting common-modal and differential-modal features to further improve the representation capability of the fused features. Extensive experiments have been conducted to validate the effectiveness of our proposed CM-YOLO method. The defect detection algorithm presented in this paper demonstrates superior detection performance in real-time PCB defect detection. In future research, we will further utilize PCB defect samples collected from industrial production and continue to enhance the application of the model based on the multimodal approach, thereby improving the practicality and effectiveness of our defect detection algorithm in real-world industrial environments.

## Figures and Tables

**Table 1:** Statistics of defect categories in train and val sets.

Category	Train Samples	Test Samples
component_missing	805	177
component_shift	151	41
dirt	117	36
lifted_pin	183	42
solder_bridging	314	82

**Table 2:** Comparison of final solutions. **Bold** values indicate the best performance for each metric.

Model	Data	Precision	Recall	mAP
YOLO11	Depth	0.832	0.934	0.900
YOLO11	RGB	0.851	0.955	0.907
YOLO11-Multi	Multi	0.803	0.949	0.946
YOLO11-CASC	Multi	0.861	0.981	0.956
YOLO11-DAWF	Multi	0.855	**0.984**	0.958
**CM-YOLO**	Multi	**0.912**	**0.984**	**0.969**

**Table 3:** Comparison with other methods. **Bold** values indicate the best performance for each metric.

Model	Data	Component Missing	Solder Bridging	Lifted Pin	Component Shift	Dirt	mAP
Faster R-CNN [42]	RGB	0.948	0.966	0.223	0.966	**0.989**	0.818
Faster R-CNN [42]	Depth	0.887	0.974	0.503	0.989	0.885	0.847
SSD [43]	RGB	0.906	0.905	0.230	0.909	**0.995**	0.789
SSD [43]	Depth	0.897	0.903	0.619	**0.998**	**0.998**	0.883
YOLOv5 [44]	Depth	0.915	0.983	0.808	0.989	0.963	0.932
YOLOv5 [44]	RGB	**0.977**	**0.995**	0.754	0.976	0.991	0.939
YOLOv8 [45]	Depth	0.904	0.986	0.760	0.979	0.952	0.916
YOLOv8 [45]	RGB	**0.978**	0.993	0.610	0.993	0.978	0.911
YOLO11	Depth	0.893	0.987	0.703	0.978	0.940	0.900
YOLO11	RGB	0.974	0.984	0.599	0.993	0.984	0.907
Ablation Study on Multimodal Fusion							
YOLO11-Multi	Multi	0.972	**0.995**	0.810	0.970	0.983	0.946
YOLO11-CASC	Multi	0.954	**0.995**	0.867	0.994	0.973	0.956
YOLO11-DAWF	Multi	0.950	0.991	0.886	0.992	0.973	0.958
**CM-YOLO (ours)**	Multi	0.974	0.993	**0.913**	0.995	0.968	**0.969**
Compared with SOTA							
D-Fine [46]	RGB	0.970	0.974	0.882	**0.998**	**0.982**	0.961
D-Fine [46]	Depth	0.922	0.968	0.891	0.959	0.981	0.944
**CM-YOLO (ours)**	Multi	**0.974**	**0.993**	**0.913**	0.995	0.968	**0.969**

**Table 4:** Comparison of model inference speed, size, and parameters. **Bold** values represent the performance of our proposed method, CM-YOLO.

Model	Time (s)	Avg Time (ms/image)	FPS	Model Size (MB)	Parameters
Faster R-CNN [42]	29.00	146.46	6.83	108.33	28,369,481
YOLOv8 [45]	3.10	15.66	63.84	5.36	2,702,926
YOLO11	3.20	16.16	61.93	5.22	2,606,272
D-Fine [46]	16.15	81.57	12.26	476.55	31,252,766
**CM-YOLO (ours)**	**8.70**	**22.02**	**45.44**	**5.20**	**2,511,527**

**Table 5:** Results of five random seed experiments: precision, recall, and mAP with mean, standard deviation, and 95% confidence intervals (CI).

Experiment Number	Precision	Recall	mAP
1	0.9443	0.9660	0.9716
2	0.9174	0.9751	0.9675
3	0.9309	0.9703	0.9713
4	0.9490	0.9662	0.9721
5	0.9424	0.9670	0.9716
Mean	0.9368	0.9685	0.9708
Std	0.0114	0.0034	0.0019
95% CI	[0.9219, 0.9517]	[0.9649, 0.9722]	[0.9689, 0.9728]

**Table 6:** Experiments simulating industrial scenarios.

Model	Dataset	Precision	Recall	mAP
CM-YOLO (ours)	Multi (darkened)	0.933	0.952	0.959
CM-YOLO (ours)	Multi (with noise)	0.880	0.934	0.946

## Data Availability

The data supporting the findings of this study are available from the corresponding author upon reasonable request.

## References

[1] Tang J., Wang Z., Zhang H., Li H., Wu P., Zeng N. (2024). A lightweight surface defect detection framework combined with dual domain attention mechanism. Expert Syst. Appl..

[2] Natarajan S., Sathaye A., Oak C., Chaplot N., Banerjee S. DEFCON: Defect Acceleration through Content Optimization. Proceedings of the 2022 IEEE International Test Conference (ITC).

[3] Wang Z., Yuan H., Lv J., Liu C., Xu H., Li J. (2024). Anomaly Detection and Fault Classification of Printed Circuit Boards Based on Multimodal Features of the Infrared Thermal Imaging. IEEE Trans. Instrum. Meas..

[4] Yu X., Lyu W., Zhou D., Wang C., Xu W. (2022). ES-Net: Efficient Scale-Aware Network for Tiny Defect Detection. IEEE Trans. Instrum. Meas..

[5] Liu X. (2024). An Adaptive Defect-Aware Attention Network for Accurate PCB-Defect Detection. IEEE Trans. Instrum. Meas..

[6] Bai X., Wang X., Liu X., Liu Q., Song J., Sebe N., Kim B. (2021). Explainable deep learning for efficient and robust pattern recognition: A survey of recent developments. Pattern Recognit..

[7] Quan Y., Chen Y., Shao Y., Teng H., Xu Y., Ji H. (2021). Image denoising using complex-valued deep CNN. Pattern Recognit..

[8] Bai T., Luo J., Zhou S., Lu Y., Wang Y. (2024). Vehicle-Type Recognition Method for Images Based on Improved Faster R-CNN Model. Sensors.

[9] Pan K., Hu H., Gu P. (2023). WD-YOLO: A More Accurate YOLO for Defect Detection in Weld X-ray Images. Sensors.

[10] Wu J., Zhou W., Qiu W., Yu L. (2022). Depth Repeated-Enhancement RGB Network for Rail Surface Defect Inspection. IEEE Signal Process. Lett..

[11] Zhou W., Hong J. (2023). FHENet: Lightweight Feature Hierarchical Exploration Network for Real-Time Rail Surface Defect Inspection in RGB-D Images. IEEE Trans. Instrum. Meas..

[12] Gao C., Chen X., Zhou J., Wang J., Shen L. (2025). Open-Set Fabric Defect Detection With Defect Generation and Transfer. IEEE Trans. Instrum. Meas..

[13] Liu Y., Gao C., Song B., Liang S. A Surface Defect Detection Algorithm for PCB Based on MobileViT-YOLO. Proceedings of the 2023 China Automation Congress (CAC).

[14] Feng B., Cai J. (2023). PCB Defect Detection via Local Detail and Global Dependency Information. Sensors.

[15] Zeng N., Wu P., Wang Z., Li H., Liu W., Liu X. (2022). A Small-Sized Object Detection Oriented Multi-Scale Feature Fusion Approach With Application to Defect Detection. IEEE Trans. Instrum. Meas..

[16] Luo J., Yang Z., Li S., Wu Y. (2021). FPCB Surface Defect Detection: A Decoupled Two-Stage Object Detection Framework. IEEE Trans. Instrum. Meas..

[17] Luo W., Luo J., Yang Z. FPC surface defect detection based on improved Faster R-CNN with decoupled RPN. Proceedings of the 2020 Chinese Automation Congress (CAC).

[18] Ling Q., Isa N.A.M., Asaari M.S.M. (2024). SDD-Net: Soldering defect detection network for printed circuit boards. Neurocomputing.

[19] Tang J., Liu S., Zhao D., Tang L., Zou W., Zheng B. (2023). PCB-YOLO: An improved detection algorithm of PCB surface defects based on YOLOv5. Sustainability.

[20] Du B., Wan F., Lei G., Xu L., Xu C., Xiong Y. (2023). YOLO-MBBi: PCB surface defect detection method based on enhanced YOLOv5. Electronics.

[21] Woo S., Park J., Lee J.-Y., Kweon I.S., Ferrari V., Hebert M., Sminchisescu C., Weiss Y. (2018). CBAM: Convolutional block attention module. Computer Vision—ECCV 2018.

[22] Li P., Xu F., Wang J., Guo H., Liu M., Du Z. (2024). DGConv: A Novel Convolutional Neural Network Approach for Weld Seam Depth Image Detection. Comput. Mater. Contin..

[23] Hwang S., Park J., Kim N., Choi Y., Kweon I.S. Multispectral pedestrian detection: Benchmark dataset and baseline. Proceedings of the IEEE Conference on Computer Vision and Pattern Recognition.

[24] Liu J., Zhang S., Wang S., Metaxas D.N. (2016). Multispectral deep neural networks for pedestrian detection. arXiv.

[25] Park K., Kim S., Sohn K. (2018). Unified multi-spectral pedestrian detection based on probabilistic fusion networks. Pattern Recognit..

[26] Li C., Song D., Tong R., Tang M. (2019). Illumination-aware faster R-CNN for robust multispectral pedestrian detection. Pattern Recognit..

[27] Zhang H., Fromont E., Lefevre S., Avignon B. Guided attentive feature fusion for multispectral pedestrian detection. Proceedings of the IEEE Winter Conference on Applications of Computer Vision.

[28] Dai W., Mujeeb A., Erdt M., Sourin A. (2020). Soldering defect detection in automatic optical inspection. Adv. Eng. Inform..

[29] Sezer A.D., Altan A. (2021). Detection of solder paste defects with an optimization-based deep learning model using image processing techniques. Solder. Surf. Mt. Technol..

[30] Xiao Y., Tian Z., Yu J., Zhang Y., Liu S., Du S., Lan X. (2020). A review of object detection based on deep learning. Multimedia Tools Appl..

[31] Tang S., He F., Huang X., Yang J. (2019). Online PCB Defect Detector on A New PCB Defect Dataset. arXiv.

[32] Wang Z., Chen W., Li T., Zhang S., Xiong R. (2022). Improved YOLOv3 detection method for PCB plug-in solder joint defects based on ordered probability density weighting and attention mechanism. AI Commun..

[33] Redmon J., Farhadi A. (2018). YOLOv3: An incremental improvement. arXiv.

[34] Rezatofighi H., Tsoi N., Gwak J., Sadeghian A., Reid I., Savarese S. Generalized intersection over union: A metric and a loss for bounding box regression. Proceedings of the 2019 IEEE/CVF Conference on Computer Vision and Pattern Recognition.

[35] Yuan M., Zhou Y., Ren X., Zhi H., Zhang J., Chen H. (2024). YOLO-HMC: An Improved Method for PCB Surface Defect Detection. IEEE Trans. Instrum. Meas..

[36] Zheng Y., Izzat I.H., Ziaee S. (2019). GFD-SSD: Gated fusion double SSD for multispectral pedestrian detection. arXiv.

[37] Zhang L., Liu Z., Zhang S., Yang X., Qiao H., Huang K., Hussain A. (2019). Cross-modality interactive attention network for multispectral pedestrian detection. Inf. Fusion.

[38] Li C., Song D., Tong R., Tang M. Multispectral pedestrian detection via simultaneous detection and segmentation. Proceedings of the British Machine Vision Conference.

[39] Li H., Wu X.-J. (2019). DenseFuse: A Fusion Approach to Infrared and Visible Images. IEEE Trans. Image Process..

[40] Zhang Y., Liu Y., Sun P., Yan H., Zhao X., Zhang L. (2020). IFCNN: A general image fusion framework based on convolutional neural network. Inf. Fusion.

[41] Lu Y., Wu Y., Liu B., Zhang T., Li B., Chu Q., Yu N. Cross-Modality Person Re-Identification With Shared-Specific Feature Transfer. Proceedings of the 2020 IEEE/CVF Conference on Computer Vision and Pattern Recognition (CVPR).

[42] Ren S., He K., Girshick R., Sun J. (2017). Faster R-CNN: Towards real-time object detection with region proposal networks. IEEE Trans. Pattern Anal. Mach. Intell..

[43] Liu W., Anguelov D., Erhan D., Szegedy C., Reed S.E., Fu C.-Y., Berg A.C. SSD: Single shot multibox detector. Proceedings of the European Conference Computer Vision.

[44] Jocher G., Stoken A., Borovec J., Changyu L., Hogan A., Diaconu L., Ingham F., Poznanski J., Fang J., Yu L. (2020). ultralytics/yolov5: V3.1-Bug fixes and performance improvements. Zenodo.

[45] Jocher G., Chaurasia A., Qiu J. Ultralytics YOLO (Version 8.0.0) [Computer software]. https://github.com/ultralytics/ultralytics.

[46] Peng Y., Li H., Wu P., Zhang Y., Sun X., Wu F. (2024). D-FINE: Redefine Regression Task in DETRs as Fine-grained Distribution Refinement. arXiv.

[47] Devaguptapu C., Akolekar N., Sharma M.M., Balasubramanian V.N. Borrow from anywhere: Pseudo multi-modal object detection in thermal imagery. Proceedings of the 2019 IEEE/CVF Conference on Computer Vision and Pattern Recognition Workshops (CVPRW).

